# The Anti-AGEing and RAGEing Potential of Isothiocyanates

**DOI:** 10.3390/molecules29245986

**Published:** 2024-12-19

**Authors:** Bradley A. Krisanits, Bhoomika Kaur, Jed W. Fahey, David P. Turner

**Affiliations:** 1 Department of Surgery, School of Medicine, Virginia Commonwealth University, Richmond, VA 23284, USA; bradley.krisanits@vcuhealth.org (B.A.K.); bhoomika.kaur7@gmail.com (B.K.); 2 Massey Cancer Center, Virginia Commonwealth University, Richmond, VA 23284, USA; 3 Departments of Medicine, Pharmacology & Molecular Sciences, Psychiatry & Behavioral Sciences, and iMIND Hopkins, The Johns Hopkins University School of Medicine, Baltimore, MD 21205, USA; jfahey@jhmi.edu; 4 Institute of Medicine, University of Maine, Orono, ME 04469, USA

**Keywords:** isothiocyanate, advanced glycation end products, receptor for advanced glycation end products, chronic disease, inflammation, oxidative stress, sulforaphane, phenethyl isothiocyanate, allyl isothiocyanate, benzyl isothiocyanate, lifestyle

## Abstract

Isothiocyanates (ITCs), found in edible plants such as cruciferous vegetables, are a group of reactive organo-sulfur phytochemicals produced by the hydrolysis of precursors known as glucosinolates. ITCs have been studied extensively both in vivo and in vitro to define their therapeutic potential for the treatment of chronic health conditions. Therapeutically, they have shown an intrinsic ability to inhibit oxidative and inflammatory phenotypes to support enhanced health. This review summarizes the current evidence supporting the observation that the antioxidant and anti-inflammatory activities of ITCs temper the pathogenic effects of a group of reactive metabolites called advanced glycation end products (AGEs). AGE exposure has significantly increased across the lifespan due to health risk factors that include dietary intake, a sedentary lifestyle, and comorbid conditions. By contributing to a chronic cycle of inflammatory stress through the aberrant activation of the transmembrane receptor for AGE (RAGE), increased AGE bioavailability is associated with chronic disease onset, progression, and severity. This review debates the potential molecular mechanisms by which ITCs may inhibit AGE bioavailability to reduce RAGE-mediated pro-oxidant and pro-inflammatory phenotypes. Bringing to light the molecular impact that ITCs may have on AGE biogenesis may stimulate novel intervention strategies for reversing or preventing the impact of lifestyle factors on chronic disease risk.

## 1. Introduction

Isothiocyanates (ITCs) are abundant naturally occurring molecules that have numerous biological activities. Molecular studies supported by pre-clinical and clinical assessments demonstrate that ITCs possess inherent antibacterial, antioxidant, and anti-inflammatory functions that may serve to prevent and/or treat chronic health conditions [1,2,3]. ITC anti-inflammatory potential is largely conferred through their ability to increase the transcriptional activation of antioxidant enzymes that inhibit inflammatory processes to restore metabolic balance [1,2]. Due to multidisciplinary research, progress has been made in understanding the molecular underpinnings that confer the anti-inflammatory potential of ITCs.

There is growing evidence to support that the antioxidant and anti-inflammatory activities of ITCs influence the accumulation and pathogenicity of a heterogenous group of lifestyle-associated reactive metabolites called advanced glycation end products (AGEs). As the final and irreversible consequence of non-enzymatic glycoxidation (the Maillard reaction), AGE adducts form on biological macromolecules and accumulate in tissues and organs over time, contributing to structural and functional degeneration [4,5,6]. AGE-bound substrates serve as high affinity ligands to the receptor for advanced glycation end products (RAGE or AGER). RAGE function is a key regulator of immune, metabolic, and oxidative pathways, and aberrant activation of RAGE is associated with unchecked chronic inflammation, contributing to chronic disease onset and complications [7,8].

**Figure 1:**
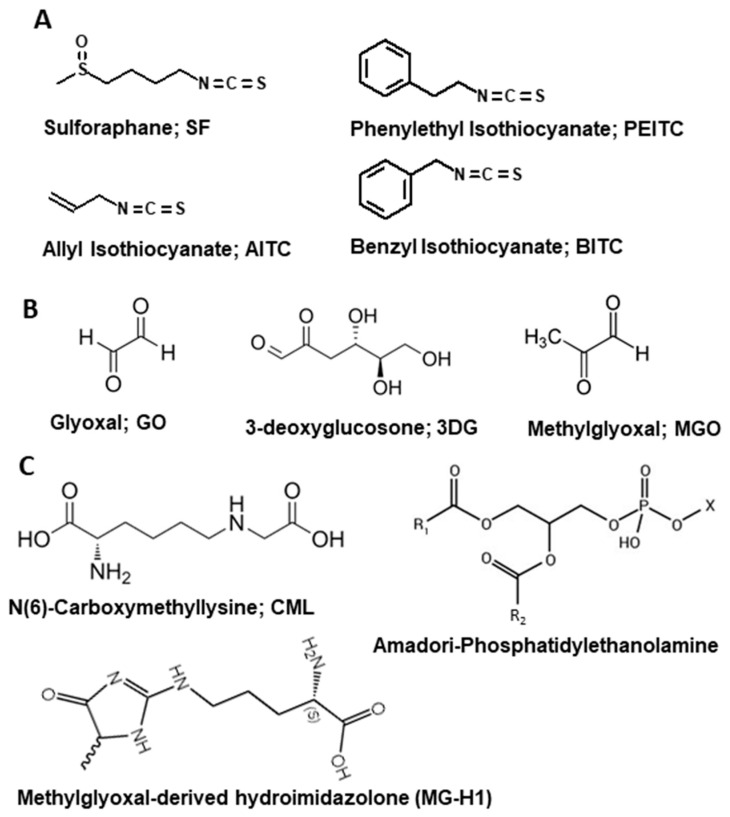
Chemical structures of relevant ITCs (**A**), reactive carbonyls (**B**), and AGE adducts (**C**).

Increased chronic disease burden, especially in developed countries, is recognized as being driven in no small part by lifestyle-associated risk factors that promote persistent inflammation [9,10]. ITC therapeutic potential against AGEs is significant because we are now being exposed to increasing levels of AGE metabolites due to lifestyle risk factors that include, but are not limited to, food intake, sedentary habits, the built environment, and disease-related comorbidities. This review focuses on four specific ITCs associated in the literature with AGE-RAGE inhibition (Figure 1A). It discusses how ITCs represent a novel therapeutic approach with which to address the influence of AGE-RAGE-mediated inflammatory stress on chronic disease risk. It discusses how the mechanisms responsible for the inherent antioxidant and anti-inflammatory activities of ITCs may disrupt a persistent cycle of AGE-RAGE-induced oxidative and metabolic dysfunction which can be exacerbated by lifestyle risk factors that lead to chronic inflammatory stress.

## 2. The Anti-Inflammatory Potential of Isothiocyanates

### 2.1. Isothiocyanates

ITCs are found within plants as water-soluble non-reactive glucosinolate precursors which comprise β-thioglucoside N-hydroxysulfates and are defined by one specific side chain and a sulfur-linked β-D-glucopyranose moiety. There are ~120 naturally occurring glucosinolate side chains (e.g., aryl-, alkyl-, indole-, benzoate-, and glycosylated) that are distributed across 16 plant families and encompass hundreds of different species [11,12]. Most notably, cruciferous vegetables such as broccoli, brussels sprouts, cabbage, and cauliflower contain multiple glucosinolates, but are often characterized by one dominant type. Glucosinolate precursors are converted to ITCs by enzymatic hydrolysis via myrosinase (a β-thioglucosidase). Myrosinase is released upon the physical disruption of plant cells due to factors such as microbial or insect attack. It is found within the human gut microbiome after the ingestion of cruciferous vegetables, resulting in the conversion of glucosinolates to ITCs. While physical disruption can lead to the activation of myrosinase, myrosinase itself is inactivated (irreversibly denatured) by processes such as the cooking of food [11,12].

The benefits of orally delivered ITCs have been realized by delivering them directly, by supplying their precursor glucosinolates and counting on intestinal microbial myrosinase and/or plant myrosinase to convert them to ITC (if delivered as fresh, raw vegetables), or by co-delivering myrosinase in dietary supplements. The ITC, when removed from their plant sources, are by and large much less stable and have a relatively short window in which to be delivered as compared to the glucosinolates from which they are derived. This can be problematic from the perspective of conducting controlled clinical trials and supplying these compounds as supplements, but is not particularly relevant when delivering fresh foods (e.g., crucifers such as cabbage, mustard, cauliflower, broccoli sprouts, moringa, or other glucosinolate-containing vegetables). The safety, bioavailability, and pharmacokinetics of these compounds has been extensively studied (there have now been over 125 clinical studies on sulforaphane (SF) alone [13,14,15]). The so-called “double-edged sword” of up-regulating the kelch-like ECH-associated protein 1 (Keap1) and nuclear factor erythroid 2-related factor 2 (Nrf2) pathway must, however, be considered when applied to patients with tumors or other actively growing cancers. Nrf2 upregulation supports growing cells, so pharmacologic doses of Nrf2 inducers (ITCs) may not be advisable, and this is still a matter of research and discussion [16,17].

### 2.2. Isothiocyanates and Their Effects on Metabolism and Health

ITCs, and in particular SF, have been shown to have wide-ranging and manifold effects on biological functions in cell and animal models and in human clinical trials [3,13]. They have shown significant therapeutic potential through their ability to increase the transcription of antioxidant enzymes, confer antibacterial activity, and to promote anti-inflammatory responses that can ameliorate metabolic changes associated with chronic disease onset and complications [1,2]. There is abundant clinical evidence for the efficacy of ITCs in human beings with chronic disease, which have been reviewed for both SF [14] and PEITC [18]. Based primarily on their detoxification capacity (primarily via the Keap1-Nrf2 pathway) and their anti-inflammatory activities, they have broad applicability to address a variety of disease states including cancer prevention and therapy [18,19,20], neurodevelopmental and neurodegenerative conditions [3], diabetes [21], ophthalmic conditions [22], kidney disease [23], diseases of the liver [24], intestinal inflammation and GI disorders [25,26], and cardiovascular disease [27]. Clinical studies initially focused on the anti-cancer potential of ITCs. These studies support the anti-tumorigenic efficacy for ITCs in a variety of cancers, including breast, gastrointestinal, lung, gastric, and prostate cancers [28,29,30,31,32,33]. Other chronic conditions where ITCs have more recently been shown to be effective agents include cardiovascular disease and type 2 diabetes (T2D), as well as those associated with metabolic, neurological, and musculoskeletal dysregulation and the overall aging process [28,34,35,36,37,38,39,40,41,42].

### 2.3. Individual ITCs Associated with AGE-RAGE Function

#### 2.3.1. Sulforaphane (SF)

SF is the most extensively studied ITC and primarily occurs in plants in the form of its precursor glucoraphanin [11]. Glucoraphanin treatment has shown efficacy in controlling circulating low-density lipoprotein cholesterol in healthy volunteers as well as lowering the risk of stroke and cardiovascular complications [43]. The efficacy of SF has been demonstrated against several chronic conditions through pathways associated with AGE-RAGE-related stress responses within the context of diabetes, hepatocellular carcinoma, rheumatoid arthritis, age-related macular degeneration, and autism spectrum disorder [35,44,45,46,47,48,49,50,51]. This efficacy is commonly achieved through the reduction of the transcriptional activity of nuclear factor kappa-light-chain-enhancer of activated B cells (NF-κB), reducing the expression of a series of inflammatory mediators including TNFα, inducible nitric oxide synthase (iNOS), interleukin-1β (IL-1β), interleukin-17 (IL-17), interleukin-6 (IL-6), interferon-γ (IFN-γ), cyclooxygenase 2 (COX-2), prostaglandins, and nitric oxide [44,45,46,47,48]. For example, the administration of broccoli sprout powder high in SF led to a reduction in diabetes-related complications such as retinal photoreceptor cell degeneration and cardiovascular damage, while improving insulin sensitivity, lipid profiles, and serum glucose levels [35]. SF influences pathways associated with AGE-RAGE-related stress responses within the context of diabetes, hepatocellular carcinoma, rheumatoid arthritis, age-related macular degeneration, and autism spectrum disorder [35,49,50,51,52,53,54,55,56,57].

#### 2.3.2. Phenethyl Isothiocyanate (PEITC)

PEITC is predominantly found as its precursor gluconasturtiin in watercress, wasabi, and garden cress [11]. PEITC was the first ITC to be studied clinically. Since being introduced in clinical research, studies have largely been limited to its assessments of cancer, DNA damage, and liver toxicity [33,38,39,58,59]. PEITC has been shown to inhibit tumor progression in models of osteosarcoma, breast, cervical, lung, colon, brain, and prostate cancer by impacting oxidative stress, inflammation, and cellular signaling [42,60,61]. PEITC has been shown to influence AGE-RAGE-mediated stress responses within the context of diabetes and cancer [53,55]. PEITC demonstrates its anti-inflammatory process in the abovementioned disorders via reducing NF-κB, focal adhesion kinase (FAK), extracellular signal-regulated kinase 1 (ERK), protein kinase B (Akt), heat shock response, and mitogen-activated protein kinases (MAPK) [61,62,63]. PEITC is also associated with increased transcriptional activation of Nrf2, positively influencing glucose metabolism and insulin sensitivity, and accompanied by reduced oxidative stress and glycolysis [31,64].

#### 2.3.3. Allyl Isothiocyanate (AITC)

AITC occurs naturally as its precursor sinigrin and contributes to the pungent tastes of mustard, radish, horseradish, and wasabi [11]. While interest is growing, limited studies have focused on AITC. However, AITC has been evaluated as a possible treatment for respiratory diseases like chronic obstructive pulmonary disease (COPD) and asthma, through its effects on the inflammatory response and increased expression of multidrug resistance-associated protein 1 (MRP1) in the lungs [56,57,58]. AITC is also associated with reduced inflammation in diseases such as osteoarthritis, inflammatory bowel disease, and obesity-induced inflammation, contributing to the activation of the transient receptor potential ankyrin 1 (TRPA1), ERK signaling, and the inhibition of monocyte chemoattractant protein-1 (MCP-1) [59,60,61]. However, to our knowledge, direct associations between AITC and AGE biogenesis have not yet been defined.

#### 2.3.4. Benzyl Isothiocyanate (BITC)

Glucotropaeolin, the precursor of BITC, is the predominant glucosinolate in garden cress, red cabbage, and papaya [11]. Of the four main ITCs discussed within this review, BITC is one of the least studied, with research largely restricted to in vitro cancer-related studies. BITC has been shown to promote G(2)/M phase arrest and subsequent apoptosis in melanoma, osteogenic sarcoma, and prostate cancer experimental models through a reduction in cyclin A, cyclin D1, cyclin-dependent kinase 2, and inhibition of DNA damage and Aurora A activity [65,66,67]. The efficacy of BITC is associated with its anti-inflammatory and antioxidant capabilities and its influence on cell cycle control, mitochondria function, and apoptosis, as well as its anti-bacterial and fungicidal properties, all of which are associated with reducing chronic inflammation in cells and tissues. However, to our knowledge, direct associations between BITC and AGE biogenesis have not been defined.

## 3. Advanced Glycation End Products (AGEs) Fuel Inflammatory Stress

### 3.1. Non-Enzymatic Glycoxidation and AGE Formation

AGEs are a highly heterogenous group of molecules that represent the final irreversible product of non-enzymatic glycoxidation (also called the Maillard reaction). This process is a complex series of chemical reactions which occurs when reactive carbonyls on oxidative and metabolic intermediates spontaneously react with electrophilic amines on biological macromolecules, leading to the reversible formation of Schiff’s bases and Amadori products [68,69,70]. Failure to remove or neutralize pools of Schiff’s base and Amadori products generates reactive carbonyl compounds such as glyoxal (GO), 3-deoxyglucosone (3DG), and methylglyoxal (MGO) (Figure 1B). Further structural rearrangement of reactive carbonyls then leads to the irreversible formation of AGE post-translational modifications such as N(6)-carboxymethyllysine (CML), methylglyoxal-derived hydroimidazolone (MG-H1) and Amadori-phosphatidylethanolamine (PE) on protein, lipid, and DNA substrates (Figure 1C) [68,69,70]. Inflammatory processes that include the peroxidation of lipids, metabolism of glucose, and the activation of the polyol pathway fuel non-enzymatic glycoxidation and AGE formation by supplying a superfluous source of reactive carbonyls [8]. Considered a part of the natural process of growing older, excessive AGE formation causes the crosslinking and misfolding of biological macromolecules, promoting their accumulation in the body. This in turn leads to the functional and structural decline of cells and tissues to accelerate their biological aging. The accumulation of AGEs and their increased bioavailability negatively impacts the onset of, and complications associated with, multiple chronic conditions, including diabetes mellitus, cancer, cardiovascular disease, and neurodegenerative disorders [4,5,6,71,72,73].

### 3.2. Lifestyle-Associated AGEs

**Figure 2:**
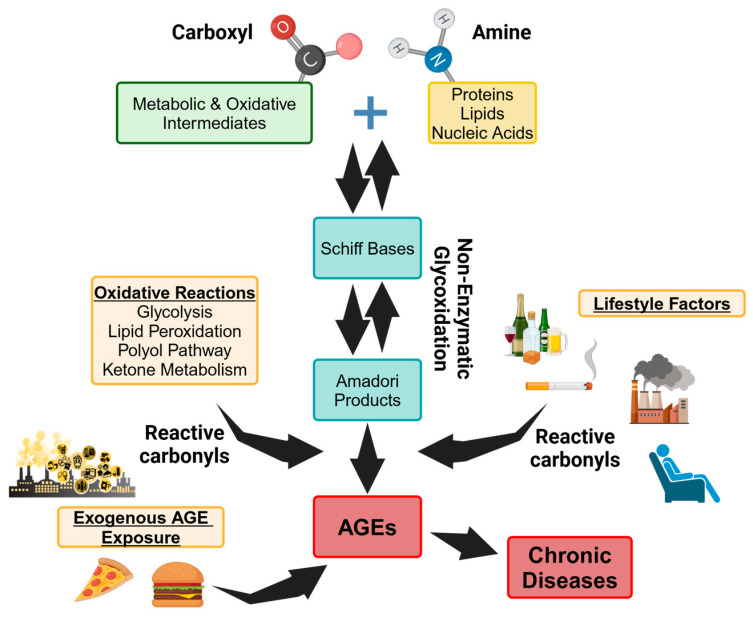
Overview of AGE-RAGE signaling and the impact of lifestyle. AGEs are formed when reactive carbonyls react with electrophilic amines in a process called non-enzymatic glycoxidation. Lifestyle factors and environmental exposures can serve to increase the available pool of reactive carbonyls needed for endogenous AGE formation and/or increase our direct exposure to pre-formed AGEs.

Largely due to modern lifestyle factors, we are now being exposed to increasing amounts of AGEs, which studies have shown contribute to greater chronic disease risk as we grow older. As reviewed, lifestyle factors such as regularly consuming an unhealthy diet high in fat or sugar, not being physically active, and being exposed to environmental exposure factors such as sunlight and pollution, can supply an additional source of reactive carbonyls that can fuel endogenous non-enzymatic glycoxidation, leading to further AGE formation [8,10,66,70,74,75]. In addition to increasing endogenous AGE formation, a significant and growing source of exogenous AGE exposure is through the consumption of heavily cooked and highly processed foods. AGE adducts are naturally present in food. However, AGE content in food is rapidly increased by high heat and pressures applied during the frying, grilling, and baking of food, as well as food manufacturing processes such as retorting and extrusion. These processes rapidly increase non-enzymatic glycoxidation rates in the food before consumption to drive AGE formation, serving as a major source of exogenous exposure [10]. By fueling non-enzymatic glycoxidation to increase both endogenous AGE formation and exogenous AGE exposure, lifestyle factors can synergize to exacerbate AGE accumulation, bioavailability, and pathogenic function (Figure 2) [8,68,69,70,72,73]. As reviewed, this is particularly relevant to underserved populations, where social, environmental, and biological factors associated with health inequity and chronic disease risk can lead to elevated AGE exposure [10,74,75]. ITCs are natural products (phytochemicals, phytonutrients, bioactives) that are present in the food supply and whose intake can be titrated simply by dietary changes (or supplementation). Thus, besides their applicability as supplements for the wealthy countries of the world, this strategy has global utility in underserved populations and lesser-developed countries, where “drugging” diseases is not a realistic strategy. While assessed in a limited cohort, AGE levels in clinical specimens of prostate cancer were found to be higher in tissue and serum specimens from African American patients when compared with European American patients [76].

### 3.3. The Receptor for Advanced Glycation End Products (RAGE)

**Figure 3:**
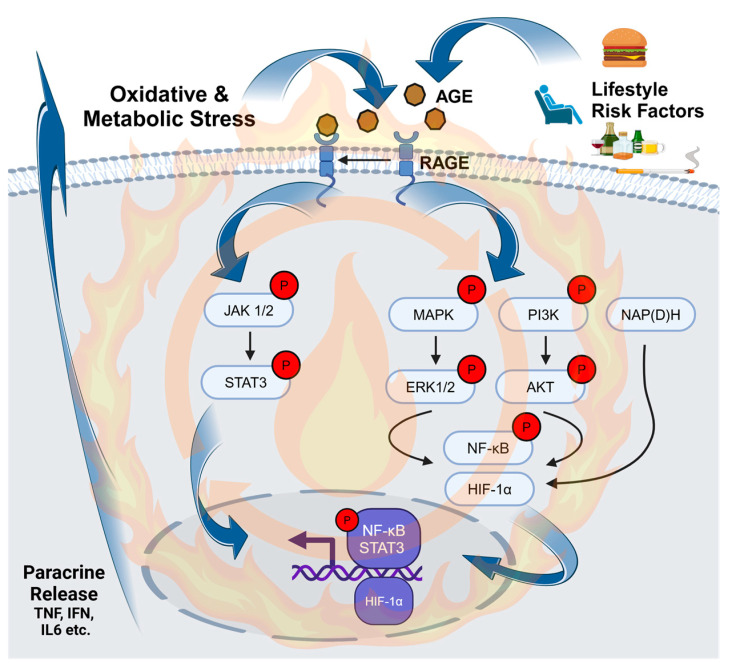
Overview of AGE-RAGE-mediated dysfunction. Lifestyle factors can combine to increase AGE bioavailability, leading to the activation of RAGE signaling pathways. This leads to the upregulation of key transcriptional regulators involved in metabolic and oxidative dysfunction, predominantly through paracrine signaling. In a persistent feed-forward loop, increased metabolic and oxidative stress can lead to further AGE formation, RAGE activation, and chronic inflammation. P = phosphorylation.

Mechanistically, AGE substrates serve as high-affinity ligands to RAGE, a pattern recognition receptor and a member of the immunoglobulin superfamily [7,68]. RAGE activation by AGE can promote paracrine-mediated oxidative and metabolic stress in multiple cell types and tissues to promote chronic inflammation (Figure 3). Physiologically, while RAGE expression is generally restricted in cells, its expression in immune cells orchestrates the host immune defense [7,68]. When bound by AGE, aberrant RAGE signaling leads to inflammatory phenotypes through the activation of multiple signaling cascades such as phosphatidylinositol-3-kinases and MAPK. This leads to the nuclear activation of master transcriptional regulators including NF-κB, the signal transducer activator of hypoxia-inducible factor 1, and STAT3 (Figure 3) [7,73]. The downstream consequence of aberrant RAGE activation is the increased paracrine release of growth factors, adhesive molecules, and cytokines/chemokines with functional roles in metabolic dysfunction, oxidative stress, and ultimately inflammation [7,68,69,73,77,78].

### 3.4. The AGE-RAGE Inflammatory Cycle

By contributing to both endogenous AGE formation and their exogenous exposure, lifestyle choices serve to exacerbate a persistent cycle of aberrant AGE-RAGE-mediated inflammatory stress [8,73,77,78]. Aberrant RAGE activation is associated with disease-associated metabolic, oxidative, and inflammatory dysfunction. By increasing AGE bioavailability. lifestyle risk factors may exacerbate RAGE-mediated dysfunction and consequently lead to further aberrant increases in metabolic, oxidative, and inflammatory stress. This in turn perpetuates the inflammatory cycle by generating additional reactive carbonyls that fuel non-enzymatic glycoxidation to further increase AGE formation. By exacerbating a persistent cycle of aberrant RAGE-mediated inflammatory stress, lifestyle-associated increases in AGE bioavailability may contribute to the current epidemics in chronic disease burden [4,5,6,7,8,65,70,77,78].

## 4. ITCs Influence AGE-RAGE Function

### 4.1. Molecular Impact of ITCs on AGE Homeostasis

**Figure 4:**
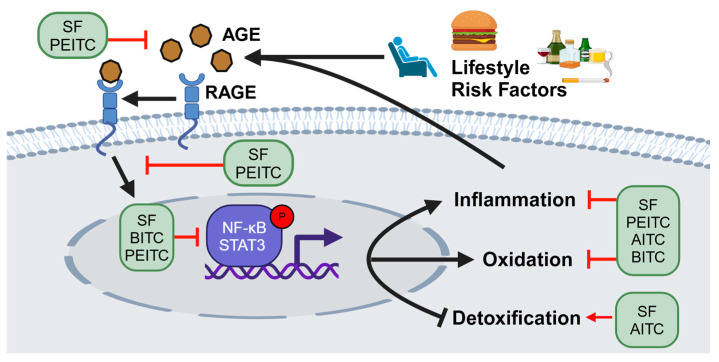
ITCs may influence lifestyle-associated AGE function by inhibiting RAGE binding and downstream effector function to inhibit oxidative and inflammatory pathways while increasing detoxification pathways associated with AGE formation.

Increasing evidence supports that the inherent anti-inflammatory potential of ITCs may negate AGE-RAGE pathogenic functions, and as such may represent a novel therapeutic approach to prevent and/or treat multiple chronic conditions [61,63,79]. The literature defines two predominant pathways for how ITCs can reduce AGE-mediated inflammation (Figure 4). First, rates of non-enzymatic glycoxidation are governed by the available pool of carbonyl precursors. By tempering the activation of metabolic and oxidative stress pathways, ITCs may serve to reduce the reactive carbonyl pool that fuels non-enzymatic glycoxidation to inhibit Schiff’s base and Amadori product generation and prevent AGE formation. Second, AGE homeostasis is maintained at least in part by detoxification enzymes that catalyze the formation of lactic acid via the condensation of reactive carbonyls. ITCs have been shown to increase the expression of detoxification enzymes such as glyoxalase 1 (GLO1) to reduce AGE bioavailability in diabetic models, which was associated with decreased RAGE function as well as oxidative stress [80].

Two often interrelated molecular mechanisms have emerged that confer the anti-inflammatory effects of ITCs. Nrf2 is a redox regulator with contrasting roles in conferring disease phenotypes. In cancer, for example, Nrf2 can function as either a tumor suppressor or a proto-oncogene, depending on cell context and the available prevailing environment. Nrf2 plays a key role in the regulation of antioxidant reactions [81]. It has been shown to inhibit AGE-mediated increases in fibronectin and TGF-β1 in glomerular mesangial cells to play a role in diabetic neuropathy [82]. Interestingly, Nrf2 itself is heavily glycated, which promotes Keap1-mediated Nrf2 degradation. Within the context of cancer, Nrf2 deglycation has been shown to be dependent upon the action of fructosamine-3-kinase [83]. NF-κβ is a key regulatory pathway inhibited by ITCs. Inhibition of NF-κβ by ITCs leads to a reduction in the secretion of pro-inflammatory cytokines such as tumor necrosis factor α (TNFα), interferon-α, IL-6, and IL-1β [79,84,85]. Associating these two regulatory pathways, ITC-mediated Nrf2 inhibition of NF-κβ has been shown to occur by two potential mechanisms: (1) inhibition of oxidative stress-mediated NF-κB activation, and (2) inhibition of NF-κβ activation through the upregulation of nuclear factor of kappa light polypeptide gene enhancer in B-cells inhibitor alpha (IκB-α) [84,85,86]. Critically, activation of NF-κB transcriptional function is a common mechanism conferring the pathogenic consequences of AGE-RAGE signaling in chronic conditions that include diabetes, cancer, cardiovascular disease, chronic kidney disease, neurodegenerative diseases, liver disease, and eye degeneration [7,8,73,78].

### 4.2. Molecular Influence of ITCs on AGE-RAGE Function

#### 4.2.1. Inflammation

While AGEs are negatively associated with multiple chronic diseases, T2D is regarded as the archetypical AGE-associated disease due to its intrinsic association with insulin resistance (IR) and hyperglycemia, both of which provide carbonyls for non-enzymatic glycoxidation. Aberrant AGE-RAGE function leading to chronic inflammation is a key event that plays a prominent role in the progression of diabetes-associated complications [53]. In contrast, in vivo and in vitro studies have shown efficacy for several ITCs in improving glucose control, endothelial function, and IR, as well as reducing diabetic complications such as vascular inflammation, neuropathy, and hepatic damage [54,87]. The reciprocal molecular connections that exist between AGEs and ITC in diabetic patients focus largely on Nrf2 regulation.

SF has been shown to produce a dose-dependent reduction in glucose production in rat hepatoma cells, which correlated with increased Nrf2 activity and the downregulation of gluconeogenetic enzymes (phospho-renolpyruvate carboxykinase 1, fructose-1,6-bisphosphatase 1, and glucose-6-phosphatase catalytic subunit) [87]. In IR human hepatocellular carcinoma cells, SF treatment also caused a dose-dependent increase in glucose uptake and alleviated IR-associated phenotypes, including glucose tolerance and insulin sensitivity [88]. Like SF, PEITC also increased the transcriptional activation of Nrf2 in adipocytes to improve glucose metabolism and insulin sensitivity [31,64]. In addition, within the context of obesity-associated IR, BITC enhanced insulin sensitivity in a Nrf2-dependent manner, lowering hyperglycemia in vivo and in vitro and potentially protecting against obesity-related T2D [89]. Linking AGEs and Nrf2, Nrf2 itself is heavily glycated, which can lead to its Keap1-mediated degradation. In nonalcoholic steatohepatitis and T2D mouse models, Nrf2 function was inhibited and degradation was increased when exposed to prolonged AGE treatment, conferred through chronic exposure to fructose [90]. This was accompanied by increased RAGE expression and pro-inflammatory nicotinamide adenine dinucleotide phosphate (NAD(P)H) oxidase activity. The direct therapeutic potential of PEITC against AGE pathogenicity was demonstrated in streptozotocin-induced diabetic Sprague Dawley rats. In this model, PEITC treatment was shown to improve renal function, restore oxidative homeostasis, inhibit NLR family pyrin domain containing 3 (NLRP3)-dependent inflammation, and suppress glycative stress in a dose-dependent manner to potentially temper the AGE-RAGE-mediated cycle of inflammatory stress [91].

Mechanistically, inflammation is often tempered by SF through the inhibition of NF-κB transcriptional activity, which reduces the expression of a series of inflammatory mediators. These mediators include oxidative and immune regulators such as TNFα, inducible nitric oxide synthase, IL-1β, interleukin-17, IL-6, IFN-γ, prostaglandins, and nitric oxide, many of which also function as AGE-RAGE signaling downstream effectors (Figure 2) [50,57,61,92]. In a direct assessment of AGE function, SF downregulated neuroinflammation caused by reactive carbonyl-induced AGE formation in BV2 microglial cells. This was once again accompanied by the inhibition of RAGE, NF-kB, and MAPK-mediated signaling [93]. These data suggest that SF may sequester reactive carbonyls, such as methylglyoxal, to temper non-enzymatic glycoxidation.

Like other ITCs, AGE inflammatory effectors that are inhibited by PEITC include NF-κB, focal adhesion kinase, ERK, protein kinase B, heat shock response, and MAPK. Specific paracrine factors reduced by PEITC inhibition of NF-κB expression include TNFα, IL-6, and IFN-γ [61,63,94]. Studies with other ITCs indicate possible associations with AGE inhibition due to common regulated pathways, but these have not been assessed for AGE-mediated affects. Treatment of Kupffer cells with BITC also showed inhibition of NLRP3 ubiquitination and subsequent activation, decreasing NLRP3-mediated inflammation and IR [94,95]. AITC has shown efficacy in the treatment of allergy-induced asthma by reducing inflammation and airway constriction through modulation of TRPA1 and ERK signaling and MCP-1 [96].

#### 4.2.2. Oxidation

AGE activation of RAGE is associated with a persistent cycle of oxidative and inflammatory stress (Section 3.3). Through the upregulation of antioxidant pathways, ITCs may serve to at least in part negate the AGE-RAGE inflammatory cycle. As for inflammatory phenotypes, Nrf2 is again assigned a central role in the antioxidant responses driven by AGE and ITC function. SF is an inducer of the cytoprotective response through Nrf2-mediated transcriptional activation of antioxidant response elements [97,98]. Associating AGEs with the same pathway, the protein deacetylase Sirtuin 1 has been shown to inhibit AGE function to activate Nrf2/ARE antioxidative pathways in glomerular mesangial cells [82]. Providing further evidence, Nrf2-mediated activation of nicotinamide adenine dinucleotide phosphate quinone oxidoreductase 1 (NQO1) was shown to represent a potential adaptive response against AGE-driven oxidative stress in diabetics [99]. Notably, SF also reduces oxidative stress through the increased activity of NQO1 [36,97,100,101,102].

Directly linking SF with AGE-RAGE signaling, AGE treatment of human umbilical endothelial cells (HUVECs), and also when injected into rat aortas, increased RAGE, oxidative stress, intercellular adhesion molecule 1, vascular cell adhesion molecule 1 (VCAM-1), and MCP-1 gene expression [103]. In the presence of SF, these AGE-mediated phenotypes were reversed; NAD(P)H oxidase and oxidative stress were reduced in HUVECS and aortic RAGE and ICAM-1 and VCAM-1 expression were reduced in rats. Of note, RAGE-Ab pretreatment following SF treatment retained gene suppression of VCAM-1, suggesting that whereas SF decreases RAGE expression, there may be additional mechanisms responsible for some AGE-mediated changes [103].

As discussed, SF directly influences AGE-RAGE-mediated stress responses within the context of diabetes [34,35,54,87,102]. The rescue of insulin sensitivity by SF, for example, may represent a form of control through the control of glucose levels, which reduces the generation of reactive metabolites that lead to AGE formation to inhibit non-enzymatic glycoxidation. In support of this premise, AGE-related changes in ferroptosis are associated with increased cardiomyopathy in diabetics. SF has been shown to inhibit AGE-mediated increases in lipid peroxidation and the expression of the ferroptosis-associated factors Ptgs2, the reactive carbonyl malondialdehyde, and MP-activated protein kinase phosphorylation, possibly via Nrf2 activation [101]. SF-treated bovine retinal pericytes in a model of diabetic retinopathy also showed reduced AGE-RAGE-mediated ROS production as well as apoptotic death compared to no treatment controls [56,104].

PEITC is also associated with the increased transcriptional activation of Nrf2 and its associated cytoprotective pathways. Nrf2 activation by PEITC was accompanied by reduced oxidative stress and glycolysis in prostate cancer cells [31,64]. PEITC was shown to function in a Nrf2-dependent manner, protecting against oxidative stress-induced IR within adipocytes (3T3-L1) by increasing the expression of anti-oxidative enzymes [64]. Potentially linking with histone glycation, PEITC also influences the epigenetic regulation of DNA methyltransferases (DNMTs) and histone deacetylases (HDACs), where reduced HDAC function has been shown to induce apoptosis and growth arrest in breast, prostate, and pancreatic cancer models [105,106].

No direct studies, to our knowledge, have assessed the therapeutic potential of AITC and BITC on AGE-RAGE function. However, AITC is also associated with reduced inflammation in diseases such as osteoarthritis and inflammatory bowel disease, and like SF, this was conferred through the activation of TRPA1, ERK signaling, and the inhibition of monocyte MCP-1 [107,108,109]. Elevated AGE levels are found in the lung, plasma, and skin of patients with chronic obstructive pulmonary disease, causing extensive tissue damage either directly or by functioning as a ligand to RAGE [110]. AITC treatment using in vivo and in vitro models of COPD was shown to reduce oxidative stress through MRP1, mediated by a reduction in AGE-RAGE-regulated IL-1β and TNFα [52,111,112,113,114,115,116,117]. AITC has also demonstrated efficacy in the treatment of allergy-induced asthma by reducing inflammation and airway constriction through modulation of TRPA1 [97]. In obesity-based IR, BITC enhanced insulin sensitivity in a Nrf2-dependent manner, lowering subsequent hyperglycemia in vivo and in vitro, potently offering protection against obese T2D [89]. BITC also directly increased the expression of Nrf2 in a model of indomethacin-induced gastric injury, in which it down-modulated the NF-κB pathway and enhanced the expression of NAD(P)H and NQO1 to control oxidative stress [118]. Treatment of Kupffer cells with BITC showed inhibition of NLRP3 ubiquitination and subsequent activation, decreasing NLRP3-mediated inflammation and IR [94,95].

#### 4.2.3. Detoxification

In addition to direct effects on oxidative pathways, the antioxidant capacity of ITCs may also be conferred by their ability to diminish the levels of reactive carbonyls through the increased activity of phase II enzymes. ITC-mediated Nrf2 activation is associated with the upregulation of phase II enzymes, including glutathione transferases, NAD(P)H:quinone reductase, epoxide hydrolase, heme oxygenase, and UDP-glucuronosyltransferase [97,98]. These enzymes function to reduce the levels of reactive carbonyls such as MGO, GO, and 3DG, which are potent AGE precursors. In cell-free assays, MGO has been shown to be metabolized by phase II enzymes in the liver through the increased transcriptional activity of Nrf2 [99]. Glo1 also sequesters reactive carbonyls to temper non-enzymatic glycoxidation [80]. Elevated levels of AGEs are found in the brains of patients with neurodegenerative disorders such as Alzheimer’s. In neuroblastoma cells, SF was shown to reverse methylglyoxal-mediated glycative damage by reducing MAPK signaling (ERK1/2, c-Jun N-terminal kinases, and p38). This was associated with the inhibition of caspase-3 activation and oxidative stress through the increased expression and activity of Glo1 [119]. Similar results were observed in primary cardiomyocytes upon SF treatment, and by SF and AITC treatment in models of hepatoma and fibroblasts, all of which induced Glo1 activity to lower reactive carbonyl levels through Nrf2 [120,121]. Assigning a further potential role for ITCs in AGE detoxification, the direct glycation of histones is associated with disease-related changes in chromatin architecture [121]. SF has been shown to reduce DNMT and HDAC activity in prostate, breast, and colon cancer cells, tempering the inflammatory environment by promoting Nrf2 expression to silence pro-inflammatory gene expression [122,123,124].

## 5. Discussion

Chronic diseases are the leading cause of death and disability within the United States, accounting for around 70% of all deaths. Six in every ten Americans (~133 million) suffer from at least one chronic disease, with one in four (over 43 million) suffering from multiple chronic conditions [125]. The increased chronic disease burden in developed countries is widely recognized as being driven in no small part by social, environmental, and biological risk factors that sustain chronic low-grade inflammation [9,10,125]. As a natural pathophysiological consequence of multiple lifestyle factors, the increased accumulation of AGEs is being increasingly recognized as an inflammatory-associated health risk factor that plays a major role in promoting multiple chronic conditions. Strategies to reduce AGE exposure across the lifespan therefore may represent a viable option to reduce chronic disease risk and outcomes in later life.

Therapeutic natural products are an alternative option to man-made drugs that may offer a more cost-effective and health-conscious option to temper or prevent chronic inflammatory phenotypes. By assigning ITCs with efficacy against the cycle of pathogenic AGE-RAGE-induced oxidative and inflammatory stress, research supports that when consumed in the diet or by supplementation, ITCs may represent a therapeutic option to address increasing AGE exposure due to the modern lifestyle. The evidence assigning ITCs with therapeutic potential against AGEs is largely inferred by their reciprocal regulation of common molecular factors involved in regulating inflammatory phenotypes and their positive and negative influence on chronic disease outcomes. While many studies are in their infancy, a growing body of evidence supports direct molecular effects for several ITCs on AGE formation, bioavailability, and or function. 

The increased formation and bioavailability of AGEs is a direct consequence of excessive carbonyl presence, often generated by metabolic and oxidative processes associated with inflammation. By their nature, the antioxidant and anti-inflammatory function of ITCs serve to reduce the pool of reactive carbonyls that fuel non-enzymatic glycoxidation to prevent AGE formation. This is conferred either through the downregulation of pro-inflammatory mediators that lead to carbonyl formation, and/or through the upregulation of detoxification enzymes responsible for the direct reversal of reactive carbonyls. This is significant because increased carbonyl formation is a direct molecular consequence of the modern lifestyle, which fuels non-enzymatic glycoxidation to increase both endogenous AGE formation and exogenous AGE exposure. As a result, this has significantly amplified our exposure to AGEs over the life course, which coincides with the epidemic increases seen in chronic disease occurrence. The antioxidative and anti-inflammatory action of ITCs may therefore serve to inhibit the persistent cycle of AGE-RAGE-mediated metabolic and oxidative stress exacerbated by reactive carbonyls, accumulated due to lifestyle-associated health risk factors.

## 6. Conclusions

We have presented a large body of evidence, both directly and by association, implicating ITCs as having therapeutic potential against AGE biogenesis and inflammatory phenotypes. Specifically, a search of the literature defined inflammation, oxidative stress, and metabolite detoxification as key drivers of disease onset and progression for multiple chronic conditions that are reciprocally regulated by ITCs and AGEs, possibly through molecular effects on NRF2 and NF-kB function. Further research is needed to confirm these direct associations between ITCs and AGE biogenesis, as well as identify new molecular associations within multiple cell types, tissues, and disease types. In addition, however, it is critical to acknowledge that there are a lack of clinical trials that directly assess the ability of ITCs to reduce AGE biogenesis and pathogenic function in humans, which are needed to confirm the true efficacy of ITCs against AGE biogenesis. One goal of this review was to bring the therapeutic relationships that exist between ITCs and AGEs to the attention of the scientific community, in order to generate interest in conducting such trials.

In particular, finely targeted studies and clinical trials that define the ability of ITCs to prevent or reverse the negative impact of lifestyle-associated AGEs represent a focus point for diet and supplement-based (“food as medicine”) intervention trials to reduce chronic disease outcomes.

## Data Availability

No new data were created or analyzed in this study. Data sharing is not applicable to this article.
